# Galectin-1 induces hepatocellular carcinoma EMT and sorafenib resistance by activating FAK/PI3K/AKT signaling

**DOI:** 10.1038/cddis.2015.324

**Published:** 2016-04-21

**Authors:** P-F Zhang, K-S Li, Y-h Shen, P-T Gao, Z-R Dong, J-B Cai, C Zhang, X-Y Huang, M-X Tian, Z-Q Hu, D-M Gao, J Fan, A-W Ke, G-M Shi

**Affiliations:** 1 Key Laboratory of Carcinogenesis and Cancer Invasion, Liver Cancer Institute, Zhongshan Hospital, Fudan University, Ministry of Education, Shanghai 200032, China; 2 State Key Laboratory of Oncogenes & Related Genes, Shanghai Cancer Institute, Renji Hospital, Shanghai Jiaotong University School of Medicine, Shanghai 200032, China; 3 Cancer Center, Institutes of Biomedical Sciences, Fudan University, Shanghai 200031, China

## Abstract

Galectin-1 (Gal-1) is involved in several pathological activities associated with tumor progression and chemoresistance, however, the role and molecular mechanism of Gal-1 activity in hepatocellular carcinoma (HCC) epithelial–mesenchymal transition (EMT) and sorafenib resistance remain enigmatic. In the present study, forced Gal-1 expression promoted HCC progression and sorafenib resistance. Gal-1 elevated *α*v*β*3-integrin expression, leading to AKT activation. Moreover, Gal-1 overexpression induced HCC cell EMT via PI3K/AKT cascade activation. Clinically, our data revealed that Gal-1 overexpression is correlated with poor HCC survival outcomes and sorafenib response. These data suggest that Gal-1 may be a potential therapeutic target for HCC and a biomarker for predicting response to sorafenib treatment.

Hepatocellular carcinoma (HCC) is the fifth most common cancer and third leading cause of cancer-related deaths worldwide.^1^ These dismal outcomes result from its high recurrence following curative liver resection and its notorious resistance to systemic chemotherapy.^2, 3^ In the past several decades, molecular therapies targeting signaling cascades involved in HCC development and progression have been explored, and sorafenib has been used as a first-line treatment for advanced HCC patients.^4^ Problematically, clinical trials have shown that on average, these patients only experience 3 months of benefits from sorafenib treatment. Moreover, none of the tested drugs showed positive responses as either first- or second-line treatment after sorafenib resistance.^5^ Consequently, it is imperative that the HCC molecular subgroup be identified and that new treatment strategies be developed.

The epithelial–mesenchymal transition (EMT) is well known to have a pivotal role in the dissemination of malignant hepatocytes during HCC progression,^6, 7^ consequently, elucidation of the molecular mechanism underlying EMT may ultimately aid in the development of innovative therapeutic strategies against HCC.

Sorafenib, a multikinase inhibitor, has been shown to improve the overall survival (OS) of patients with advanced HCC and represents a breakthrough in the clinical management of this cancer.^8^ Although sorafenib has been shown to improve OS in large randomized Phase III studies, the response rate is actually quite low and the mean extension of survival is 3 months for advanced HCC cases.^9, 10^ Consequently, it is imperative that the molecular mechanisms involved in sorafenib resistance are identified and used to improve HCC response to sorafenib.

Galectin-1 (Gal-1), a 14.5-kDa protein, is regulated by HIF-1 and has vital protumorigenic roles within the tumor microenvironment.^11^ Dysregulation of Gal-1 expression is associated with resistance to chemotherapy through H-Ras/Raf/extracellular signal-regulated kinase (ERK) pathway activation.^12^ Gal-1 overexpression also mediates migration and invasion via increased phosphorylation of AKT, mTOR and p70 kinases in cancer cells.^13^ Moreover, sorafenib response is impaired in HCC with dysregulated p-ERK and p-AKT activation.^14, 15^

Previous studies have confirmed that integrins facilitate tumor metastasis and influence the malignant phenotypes of several cancers.^16^ Integrins are predominantly expressed in epithelial cells where they serve as laminin receptors, and the *α*v and *β*3 subunits are both upregulated in cancer progression and therapy resistance.^17, 18, 19, 20^

In this study, we found that forced Gal-1 expression induces EMT through an *α*v*β*3-integrin/FAK/PI3K/AKT signaling pathway in HCC cells. Moreover, high levels of Gal-1 are associated with impaired sorafenib response and reduced OS.

## Results

### Gal-1 is upregulated in human HCC and is correlated with poor prognosis

**Figure 1:**
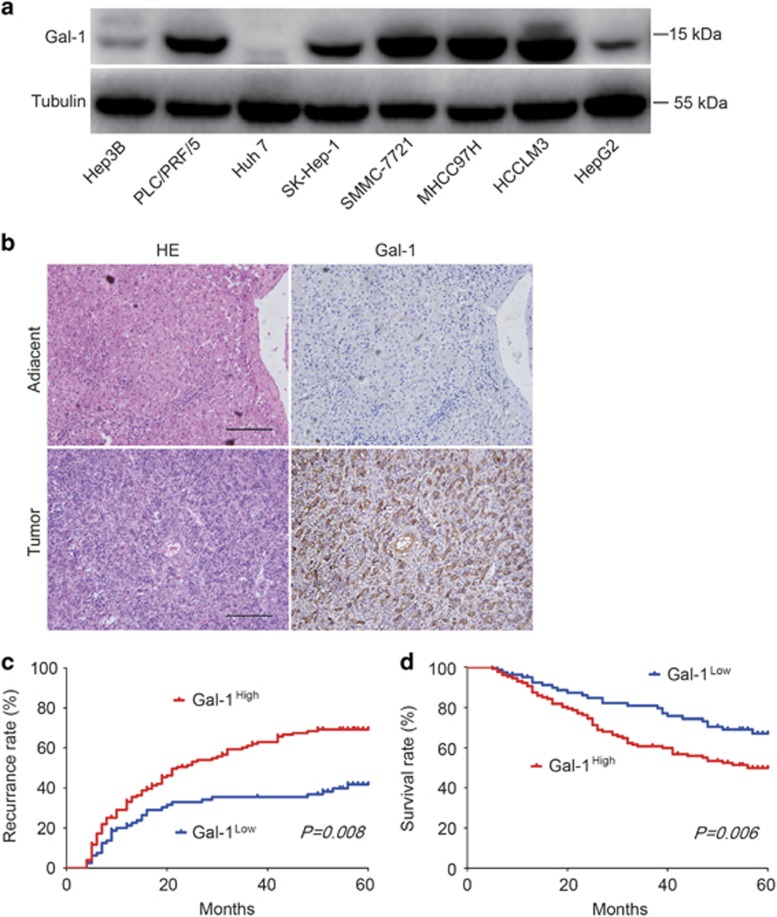
Gal-1 is overexpressed in HCC tissues and its expression is correlated with HCC clinical outcome. (**a**) Gal-1 expression in several HCC lines was examined using western blot analysis. Tubulin was used as a control for loading. (**b**) Gal-1 immunostaining using 3,3′-diaminobenzidine (DAB; brown) in HCC tissues and adjacent normal liver. (**c**,**d**) The cumulative recurrence and overall survival rates of 209 patients with HCC were compared between Gal-1-low and -high groups using Kaplan–Meier methods (log-rank test). Scale bar, 100 *μ*m

To explore the role of Gal-1 in HCC progression, we first evaluated Gal-1 expression in various human HCC cell lines. We found that Gal-1 protein expression was significantly increased in highly metastatic cell lines (MHCC97H and HCCLM3) compared with low metastatic HCC cell lines (Hep3B and Huh-7; Figure 1a). We then performed immunohistochemistry (IHC) analysis of Gal-1 expression in 209 HCC patients, finding that Gal-1 expression was significantly higher in HCC tissues compared with adjacent normal tissues (Figure 1b). For further analysis, patients were separated into groups with high (Gal-1-high, moderate and strong; *n*=128) and low Gal-1 expression (Gal-1-low, negative and weak; *n*=81) groups. There was a striking, and statistically significant inverse association between Gal-1 intensity and recurrence-free survival (RFS; *P*=0.008; Figure 1c), and to a less extent, over survival (OS) (*P*=0.006; Figure 1d). The respective median RFS and OS time for Gal-1-high patients were 40.5 and 47.7 months, compared with 53.1 and 57.8 months for Gal-1-low patients. Multivariate analysis revealed that Gal-1 intensity in tumors was an independent predictor for both RFS and OS (Supplementary Tables 2 and 3), therefore, Gal-1 expression is a valuable predictor for recurrence and survival in HCC patients.

### Gal-1 promotes HCC cell invasion *in vitro* and lung metastasis *in vivo*

**Figure 2:**
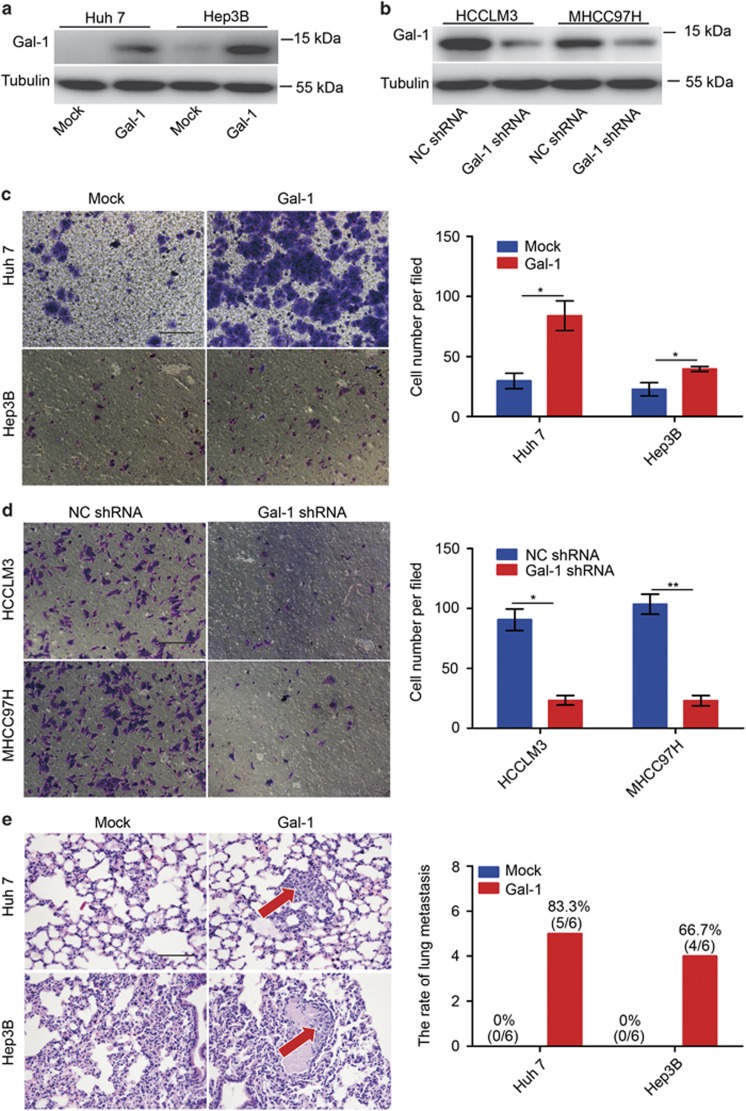
High expression of Gal-1 promotes HCC metastasis both *in vitro* and *in vivo*. (**a**,**b**) Gal-1 expression in HCC cells was modified by shRNA interference and cDNA transfection. (**c**,**d**) Cancer cell invasion was measured using transwell assays. (**e**) Representative views of lung tissue sections from each group; pulmonary metastasis rates are shown. *P<0.05, **P<0.05. Scale bar, 100 *μ*m

Western blot analysis was used to confirm stable upregulation of Gal-1 expression in Hep3B and Huh-7 cells, and Gal-1 knockdown in HCCLM3 and MHCC97H cells (Figures 2a and b). *In vitro* invasion assays revealed that the Huh-7- Gal-1 and Hep3B-Gal-1 groups had significantly larger populations of invasive cells compared with the control cells (Figure 2c). In contrast, the population of invasive cells in the HCCLM3-Gal-1 shRNA and MHCC97H-Gal-1 shRNA groups was significantly reduced compared with the control (Figure 2d). Following orthotropic transplantation of Hep3B-Gal-1, Huh-7- Gal-1 and mock cells into nude mice, all groups successfully formed liver tumors. Pulmonary metastases occurred in both the Huh-7- Gal-1 (5/6) and Hep3B-Gal-1 (4/6) groups; a representative lung metastasis is shown in Figure 2e. In contrast, pulmonary metastases were not observed in the Hep3B-mock and Huh-7-mock groups. Together, the *in vitro* and *in vivo* data show that Gal-1 significantly contributes to HCC tumor metastasis.

### Gal-1 induces *α*v*β*3-integrin expression and FAK/PI3K/AKT signaling activation

**Figure 3:**
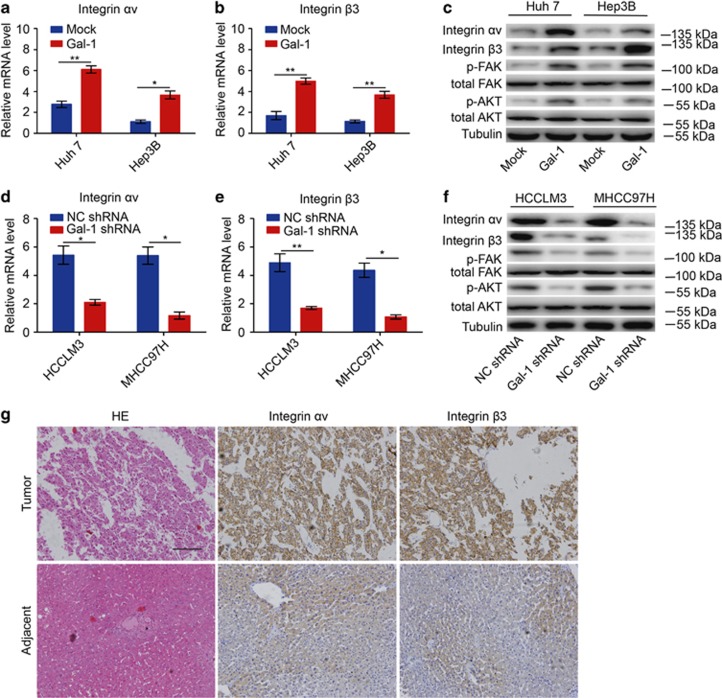
Gal-1 overexpression induces FAK/PI3K/AKT signaling hyperactivation in HCC cells. (**a**,**b**) Forced Gal-1 expression enhanced *α*v- and *β*3-integrin mRNA expression in HCC cells. (**c**) Forced Gal-1 expression increased protein expression of *α*v and *β*3 integrin, p-FAK, and p-AKT in HCC cells. (**d**,**e**) mRNA expression levels of *α*v and *β*3 integrin were reduced in HCC cells treated with Gal-1 shRNA. (**f**) Protein expression levels of *α*v and *β*3 integrin, p-FAK, and p-AKT were reduced in HCC cells treated with Gal-1 shRNA. (**g**) Immunostaining (DAB) for *α*v and *β*3 integrin in HCC and adjacent normal liver tissues. Data are represented as the mean±S.D., *n*=3. *P<0.05, and **P<0.01. Scale bar, 100 *μ*m

To characterize the molecular pathways affected by Gal-1 in HCC cells, we examined two microarray-based global gene expression profiling studies of cancer cells with endogenous forced Gal-1 levels or Gal-1 knockdown.^21, 22^ We speculated that the *α*v- and *β*3-integrin subunits may be candidate Gal-1 targets. Real-time PCR and western blot analysis confirmed that the mRNA and protein expression levels of both the *α*v- and *β*3-integrin subunits were reduced in HCCLM3 and MHCC97H cells with Gal-1 knockdown, whereas Gal-1 overexpression increased *α*v- and *β*3-integrin subunit levels in Huh-7 and Hep3B cells (Figures 3a and f). Furthermore, the phosphorylation of FAK, a downstream integrin kinase, was also increased in Huh-7 and Hep3B cells overexpressing Gal-1, while FAK activation was reduced in HCCLM3 and MHCC97H cells with Gal-1 knockdown (Figures 3e and f). The downstream FAK signaling molecule AKT was also activated in Huh-7 and Hep3B cells overexpressing Gal-1, while AKT activation was reduced in HCCLM3 and MHCC97H cells with Gal-1 knockdown (Figures 3e and f).

We then examined *α*v*β*3-integrin expression in a cohort of 209 HCC patients, finding that *α*v and *β*3-integrin subunit staining was localized to the plasma membrane (Figure 3g). We found that 89 of 209 HCC cases (42.6%) exhibited high levels of both the *α*v and *β*3-integrin subunits. Strikingly, IHC analysis showed that patients with HCC and Gal-1 overexpression tended to have higher levels of *α*v and *β*3-integrin subunits. These results indicate that Gal-1 overexpression may promote tumor progression by inducing *α*v and *β*3-integrin subunit expression in HCC.

To further explore the relationship between Gal-1, *α*v and *β*3 integrin and p-AKT, we examined tissue from 209 HCC cases for Gal-1, *α*v and *β*3 integrin, and p-AKT expression. The expression patterns of these proteins in HCC tissues revealed a positive correlation between protein levels of Gal-1 with *α*v integrin (*P*=0.0119, *R*^2^=0.2056), *β*3 integrin (*P*=0.0242, *R*^2^=0.1686) and p-AKT (*P*=0.0194, *R*^2^=0.1802; Supplementary Figure 1).

### High Gal-1 expression induces FAK and AKT hyperactivation by selectively amplifying the *α*v*β*3-integrin signal

**Figure 4:**
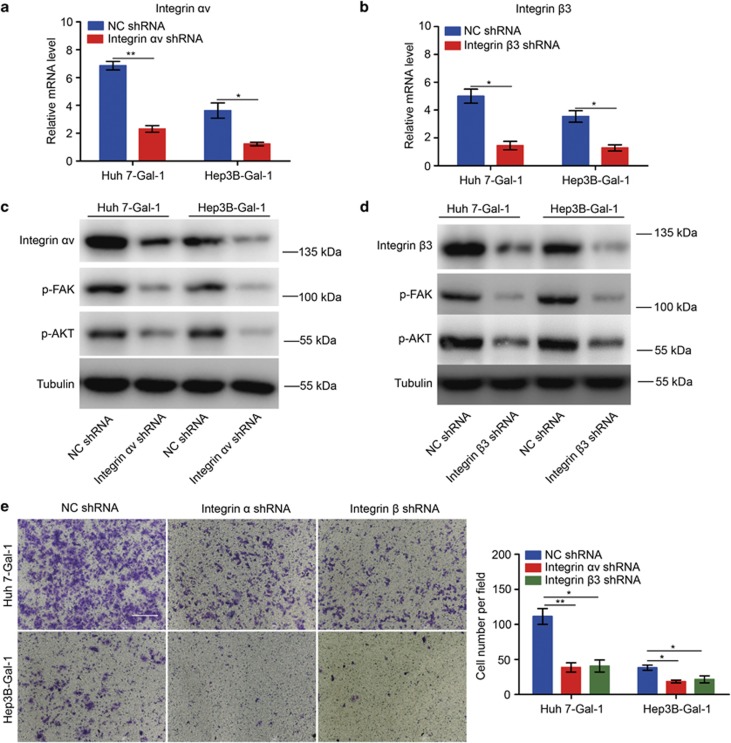
Gal-1 regulates the FAK/PI3K/AKT pathway by upregulating HCC cell expression of *α*v*β*3 integrin. (**a**,**b**) Real-time PCR revealed downregulation of the *α*v- and *β*3-integrin subunits in Huh-7- Gal-1 and Hep3B-Gal-1 cells. (**c**,**d**) Modulation of *α*v- and *β*3-integrin subunit expression reversed FAK/PI3K/AKT signaling hyperactivation in Huh-7- Gal-1 and Hep3B-Gal-1 cells. (**e**) Downregulation of the *α*v- and *β*3-integrin subunits markedly inhibits HCC cell invasion *in vitro*. Data are represented as the mean±S.D., *n*=3. *P<0.05, and **P<0.01. Scale bar, 100 *μ*m

Gal-1 increases *α*v*β*3-integrin expression and activates FAK/PI3K/AKT signaling in HCC cells, therefore, we examined whether the oncogenic effects of Gal-1 could be reversed by loss of either the *α*v- or *β*3-integrin subunits. We first transfected Huh-7- Gal-1 and Hep3B-Gal-1 cells with an *α*v-integrin shRNA plasmid and validated the shRNA knockdown efficacy using real-time PCR and western blot analysis (Figures 4a and c). Knockdown of *α*v integrin reduced Gal-1 overexpression-mediated FAK and AKT phosphorylation and invasion *in vitro* (Figures 4c and e). We then transfected Huh-7- Gal-1 and Hep3B-Gal-1 cells with a *β*3-integrin shRNA plasmid, and observed results similar to those produced by integrin *α*v knockdown (Figures 4b and e). Interestingly, knockdown of *α*v/*β*3-integrin expression was sufficient to block HCCLM3 and MHCC97H cell invasion (Supplementary Figure 2). These results indicate that Gal-1 might regulate the FAK/PI3K/AKT pathway by upregulating *α*v*β*3-integrin expression.

### Gal-1 overexpression induces HCC EMT through FAK/PI3K/AKT pathway hyperactivation

**Figure 5:**
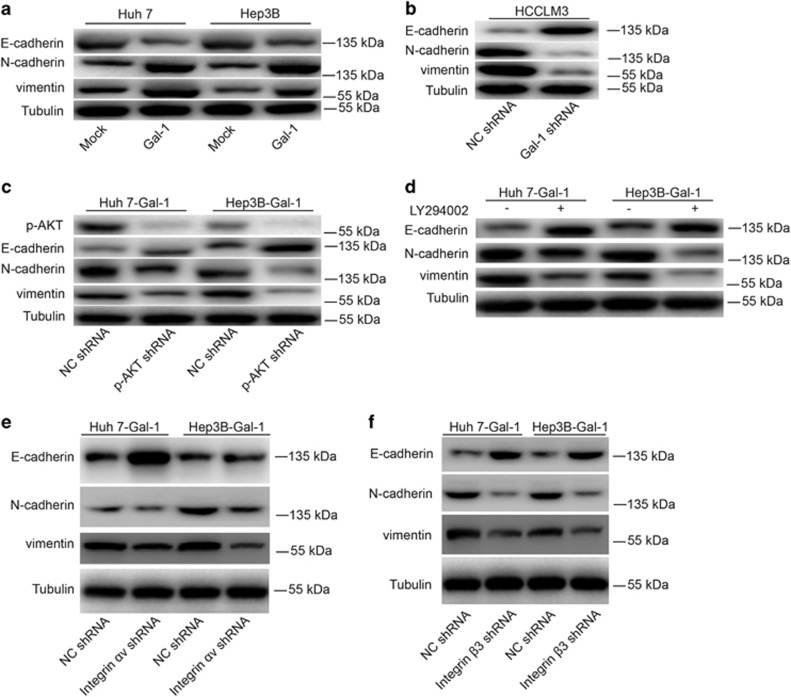
Gal-1 overexpression mediated HCC cell EMT via PI3K/AKT signaling. (**a**,**b**) EMT markers in cells expressing high and low levels of Gal-1 (Huh-7- Gal-1 *versus* Huh-7-mock, Hep3B-Gal-1 *versus* Hep3B-mock, and HCCLM3-NC shRNA *versus* HCCLM3-Gal-1 shRNA). (**c**,**d**) Modulation of AKT expression and treatment with LY294002 reversed Gal-1 overexpression-mediated EMT in Huh-7- Gal-1 and Hep3B-Gal-1 cells. (**e**,**f**) Modulation of *α*v- and *β*3-integrin expression reversed Gal-1-overexpression-mediated EMT in Huh-7- Gal-1 and Hep3B-Gal-1 cells. All experiments were repeated at least three times

On the basis of the association between Gal-1 expression and HCC progression *in vivo* and *in vitro*, and given that EMT is considered to be a striking feature of most cancers and has an important role in cancer migration and invasion, we examined the expression of epithelial and mesenchymal markers, as well as of other molecules thought to induce EMT in cancer cells. As shown in Figure 5a, Huh-7- Gal-1 and Hep3B-Gal-1 cells expressed reduced levels of the epithelial marker E-cadherin compared with Huh-7-mock and Hep3B-mock cells. In contrast, the mesenchymal markers vimentin and N-cadherin were significantly upregulated in Huh-7- Gal-1 and Hep3B-Gal-1 cells compared with Huh-7-mock and Hep3B-mock cells. HCCLM3 is a highly metastatic cell line that expresses low levels of E-cadherin and high levels of vimentin and is thus thought to present a mesenchymal-like phenotype.^23^ Interestingly, E-cadherin levels were higher in HCCLM3-Gal-1 shRNA cells than in HCCLM3-NC shRNA cells, while the mesenchymal-associated genes vimentin and N-cadherin were downregulated in HCCLM3-Gal-1 shRNA cells (Figure 5b).

A previous study reported that the hyperactivation of PI3K signaling is responsible for EMT, primarily via the PI3K/AKT/Snail/PTEN feedback loop.^6^ The results obtained thus far indicated that the FAK/PI3K/AKT signaling may mediate HCC EMT owing to high expression of Gal-1. We aimed to test this idea by downregulating endogenous AKT and inhibiting PI3K signaling using a PI3K inhibitor, LY294002 (20 *μ*mol/l). As anticipated, Huh-7- Gal-1 and Hep3B-Gal-1 cells lacking AKT (due to shRNA or inhibition of PI3K signaling) expressed high levels of E-cadherin and low levels of vimentin and N-cadherin compared with control cells (Figures 5c and d).

Furthermore, the Huh-7- Gal-1 and Hep3B-Gal-1 cells lacking either *α*v- or *β*3-integrin expressed high levels of E-cadherin and low levels of vimentin and N-cadherin compared with control cells (Figures 5e and f).

### Gal-1 expression is inversely correlated with HCC sensitivity to sorafenib

**Figure 6:**
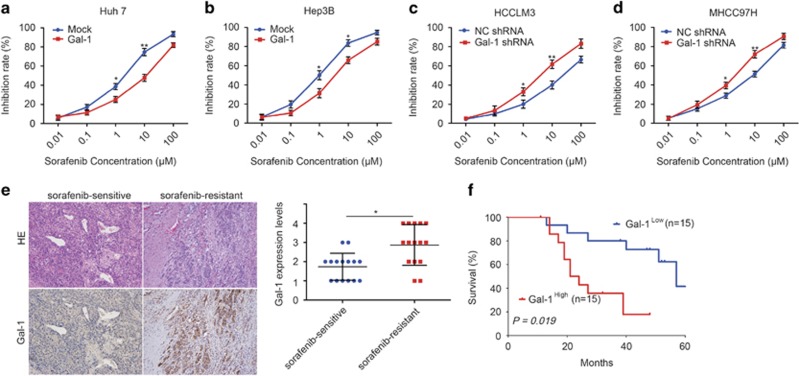
Gal-1 induces sorafenib resistance in HCC cells. (**a**,**b**) Forced Gal-1 expression in Huh-7 and Hep3B cells reduced their sensitivity to sorafenib. (**c**,**d**) Gal-1 knockdown in HCCLM3 and MHCC97H cells increased their sensitivity to sorafenib. (**e**) Gal-1 immunostaining (DAB) of sorafenib-sensitive and -resistant HCC tissues. (**f**) Comparison of overall survival curves for patients with high and low Gal-1 expression that were treated with sorafenib. Data are represented as the mean±S.D., *n*=3. *P<0.05, and **P<0.01. Scale bar, 100 *μ*m

To examine the role of Gal-1 in HCC cell sensitivity to sorafenib, we generated Huh-7 and Hep3B cells overexpressing Gal-1. For Huh-7- Gal-1 and Huh-7-mock cells, the sorafenib IC_50_ values were 7.12±0.72 *μ*m and 1.52±0.40 *μ*m, respectively (*P*<0.05; Figure 6a); for Hep3B-Gal-1 and Hep3B-mock cells, the sorafenib IC_50_ values were 3.65±0.77 *μ*m and 0.92±0.23 *μ*m, respectively (*P*<0.05; Figure 6b). Together, these data suggest that Gal-1 overexpression leads to comparative HCC cell resistance to sorafenib. To further elucidate the role of Gal-1 in HCC cell resistance to sorafenib, HCCLM3 and MHCC97H cells, which exhibit high levels of Gal-1 protein expression, were treated with Gal-1 shRNA. For HCCLM3-Gal-1 shRNA and HCCLM3-NC shRNA cells, the sorafenib IC_50_ values were 4.11±1.09 *μ*m and 22.13±2.37 *μ*m (*P*<0.05), respectively (Figure 6c); for MHCC97H Gal-1 shRNA and MHCC97H NC shRNA cells, the sorafenib IC_50_ values were 1.78±0.45 *μ*m and 5.64±0.61 *μ*m (*P*<0.05), respectively (Figure 6d).

We then analyzed retrospective data from 30 advanced recurrent HCC patients receiving combined sorafenib and transarterial chemoembolization therapy who had undergone liver resection 2−60 months before the combined therapy; patient demographics (Supplementary Table 4) and OS were recorded. Gal-1 expression levels were then measured (Figure 6e), and Kaplan–Meier survival analysis indicated that the OS probability for the Gal-1-high group was much lower than that for the Gal-1-low group (Figure 6f). The median OS was 9.0 months in the Gal-1-high group and 14.0 months in the Gal-1-low group (Gal-1-high group hazard ratio 2.879; 95% confidence interval, 1.019–6.271; *P*<0.05); therefore, we conclude that high levels of Gal-1 lead to HCC sorafenib resistance.

## Discussion

The majority of our results in this study reinforce the notion that Gal-1 is a positive regulator of HCC progression. First, Gal-1 promoted HCC invasion *in vitro* and *in vivo*; second, Gal-1 overexpression fostered HCC progression by inducing EMT; and third, forced Gal-1 expression reduced HCC sensitivity to sorafenib. We also demonstrate for the first time that Gal-1 enhanced *α*v*β*3-integrin expression, which activates FAK/PI3K/AKT signaling. Clinically, we found that Gal-1 expression was correlated with overall patient survival and disease recurrence. Moreover, we demonstrated that Gal-1 overexpression activates FAK/PI3K/AKT signaling to induce HCC EMT. The above results support the notion that Gal-1 has a vital role in HCC progression.

A recent study showed that forced Gal-1 expression resulted in enhanced H-Ras-GTP membrane association, an increased number of Raf-1 recruitment sites, triggered sustained MEK-ERK pathway activation and enhanced cell transformation.^24^ The Ras-ERK pathway is required for EMT and contributes to the maintenance of an undifferentiated/mesenchymal state in tumor cells. This pathway cooperates with other pathways to promote expression of EMT-related genes, including mesenchymal genes and transcriptional repressors of epithelial genes. Consequently, H-Ras may be a potential target against Gal-1-induced HCC progression.^25^ Moreover, Gal-1 activates NF-*κ*B in kidney cancer, inducing CXCR4 expression.^26^ The SDF-1/CXCR4 axis can trigger EMT in glioblastoma,^27^ meaning that CXCR4 could be another downstream target that mediates Gal-1-induced HCC progression; however, our results indicate that forced expression of Gal-1 in HCC cells is accompanied by an upregulation of *α*v*β*3-integrin mRNAs and proteins. Furthermore, *α*v*β*3-integrin knockdown reversed the mesenchymal phenotype conferred by Gal-1 overexpression, suggesting that Gal-1 can induce EMT by upregulating *α*v*β*3 integrin. Using gene expression analysis, we demonstrated that Gal-1 overexpression induces FAK/PI3K/AKT signaling hyperactivity by upregulating *α*v*β*3 integrin. Therefore, we conclude that *α*v*β*3 integrin has a critical role in Gal-1-induced HCC progression.

EMT is regulated by upstream pathways including PI3K/AKT, MAPK, and TGF-*β* etc.^6, 14, 28^ Emerging evidence has suggested that EMT is involved in cancer chemoresistance and that inhibiting EMT can reverse this resistance.^29, 30^ Moreover, EMT has been reported to function in HCC resistance to sorafenib^14^ and hyperactive PI3K/AKT signaling was one of the primary causes.^15, 31^ These previous studies indicate that PI3K/AKT signaling hyperactivity may function in Gal-1-induced HCC resistance to sorafenib. In the present study we showed that Gal-1 overexpression leads to comparative HCC cell resistance to sorafenib *in vitro*, furthermore, despite a small cohort, clinical data also indicate that high levels of Gal-1 lead to HCC sorafenib resistance.

In conclusion, we provide insight into the biological function of Gal-1 signaling in HCC and demonstrate that Gal-1 overexpression activates the FAK/PI3K/AKT pathway by upregulating expression of *α*v*β*3 integrin, leading to enhanced HCC invasion via EMT and sorafenib resistance. Consequently, our study suggests that targeting Gal-1 in a subset of HCCs would be an optimal therapeutic strategy and suggests that Gal-1 may be a biomarker for predicting responsiveness to sorafenib treatment.

## Materials and Methods

### Cell lines and clinical samples

The human HCC cell lines Huh-7, Hep3B, SK-Hep-1, HepG2, and PLC/PRF/5 were purchased from the American Type Culture Collection (Manassas, VA, USA), SMMC-7721 was preserved in our institute, and HCCLM3 and MHCC97H were established in the Liver Cancer Institute of the Zhongshan Hospital of Fudan University (Shanghai, China).^6^ All of the cell lines were routinely maintained.

Paraffin-embedded specimens of normal human liver and HCC tissues were obtained from the Zhongshan Hospital of Fudan University (Clinicopathological characteristics were listed in Supplementary Table 1) and used for IHC. Sample collections were performed after receiving approval from the institutional ethics review committee of the Zhongshan Hospital of Fudan University, and no patients had undergone chemotherapy before surgery. Surgical evaluation was used to determine the clinical stage and presence of metastases, and pathologists performed histopathologic analysis to assess the cancer type and grade.

### Transwell invasion assay

Cell invasion was measured using the transwell matrigel invasion assay. For Matrigel invasion assays, cells were suspended in DMEM without serum and then placed in the cell culture insert precoated with matrigel (BD Biosciences, San Jose, CA, USA). Warmed culture media containing 10% FBS was added to the well, and the cells were then incubated for 24 h at 37 °C in 5% CO_2_, fixed with 4% paraformaldehyde and stained with 0.1% crystal violet (Sigma, St. Louis, MO, USA). A light microscope was then used to count the number of cells in five randomly selected areas.

### *In vivo* tumor invasion and metastasis assays

Male athymic BALB/c nude mice were used for animal studies. An orthotopic human HCC xenograft model was established for analysis of *in vivo* tumor invasion and metastasis as described in previously.^32^ All procedures were approved by the Animal Care and Use Committee of Shanghai, China.

### Western blot analysis

Cell lysates were collected and centrifuged for 15 min at 12 000 r.p.m., 4 °C. The supernatant was transferred to a clean tube and proteins concentrations were then quantified using the BCA Kit (Pierce, Rockford, IL, USA). Proteins were then separated on SDS-PAGE gels and transferred to nitrocellulose membranes. The membranes were then blocked with 5% skim milk for 2 h at room temperature and incubated overnight at 4 °C with primary antibodies. Immune complexes were then detected by incubating the nitrocellulose membranes with HRP-conjugated goat anti-mouse/rabbit antibody (Santa Cruz, CA, USA) for 2 h at room temperature, followed by exposure of the membrane to enhanced chemiluminescence reagents (Pierce, Rockford).

### Lentivirus production and target cells transduction

The pwpt-Gal-1, pwpt-GFP, pshRNA-copGFP-Gal-1 shRNA, and pshRNA-copGFP-NC shRNA lentiviruses were produced and used for target cell transductions as described previously.^33^

### Construction of tissue microarrays and IHC

Tissue microarray and IHC staining analyses of target genes were performed as previously described.^6^ Paraffin-embedded tissue sections (4 *μ*m) were prepared using classical methods and Gal-1, and *α*v-, and *β*3-integrin expression was detected using an immunoperoxidase method. According to the intensity and total area of the staining, Gal-1, and *α*v- and *β*3-integrin expression levels were classified as either high (>20% of tumor section) or low (<20% of tumor section) using an integrated imaging system (MetaMorph Imaging System version 3.0; Universal Imaging Corp, Buckinghamshire, UK). Briefly, three representative fields were captured for each case using the Leica QWin Plus v3 software (Leica Microsystems Inc, Buffalo Grove, IL, USA) under identical settings and magnification (× 200). The integrated absorbances and areas of the photographs were measured using Image-Pro Plus v6.0 software (Media Cybernetics, Inc, Bethesda, MD, USA). A uniform setting of color segmentation setting was used for counting the integrated absorbance of each picture.

### Real-time PCR

Total RNA was extracted using the TRIzol Kit (Invitrogen, Carlsbad, CA, USA) according to the manufacturer's instructions, and real-time PCR was performed using the SYBR Green PCR kit (TaKaRa, Otsu, Japan) according to the manufacturer's instructions. *β*-actin served as an internal control.

### Statistical analysis

Statistical analysis was performed using SPSS 16.0 software (Chicago, IL, USA). All tests were two-tailed and *P*<0.05 was considered to be statistically significant.

## Abbreviations

HCC: hepatocellular carcinoma
Gal-1: galectin-1
EMT: epithelial–mesenchymal transition
HIF-1: Hypoxia-inducible factor-1
ROS: reactive oxygen species
ERK: extracellular regulated protein kinase
OS: overall survival
RFS: recurrence-free survival
PI3K: phosphatidylinositol-3-kinase
real-time PCR: real-time polymerase chain reaction
shRNA: short hairpin RNA
