# Supplementary material for: Galectin-1 induces hepatocellular carcinoma EMT and sorafenib resistance by activating FAK/PI3K/AKT signaling
Source: Cell Death Dis. 2016 Apr 21;7(4):e2201–. doi: 10.1038/cddis.2015.324 (PMC4855644; doi:10.1038/cddis.2015.324)
Supplement: Supplementary Figure Legends [file cddis2015324x1.doc]

**Supplementary Figure Legends**

**Supplementary Figure 1.** **Gal-1 expression was positively correlated with integrin αv/ integrin β3/ p-AKT expression in HCC.** (a) The expression of Gal-1, integrin αv, integrin β3, and p-AKT in 209 cases of HCC tissues were detected by IHC staining. (b-d) A positive correlation between Gal-1 and integrin αv/ integrin β3/ p-AKT was observed in tumor tissues at the protein levels. Scale bar: 100 μm.

**Supplementary Figure 2. Knockdown integrin αv or integrin β3 expression inhibits HCC invasion *in vitro*.** (a and b) Integrin αv and integrin β3 protein expression in HCC cells was modified by shRNA interference. (c) The invasion of cancer cells was measured by transwell assays. ***P<0.01*, and **P<0.05*. Scale bar: 100 μm.
