# Supplementary material for: Galectin-1 induces hepatocellular carcinoma EMT and sorafenib resistance by activating FAK/PI3K/AKT signaling
Source: Cell Death Dis. 2016 Apr 21;7(4):e2201–. doi: 10.1038/cddis.2015.324 (PMC4855644; doi:10.1038/cddis.2015.324)
Supplement: Supplementary Materials and Methods [file cddis2015324x2.doc]

**Supplementary Materials and Methods**

***The correlation analysis among Gal-1 and integrin αv/ integrin β3/ p-AKT.***

Immunohistochemical staining for the target proteins was carried out on sections of the formalin-fixed samples on the tissue microarray. A positive reaction for Gal-1, integrin αv, integrin β3, and p-AKT expression were scored in five grade categories depending on the intensity of the staining, i.e., 0, 1, 2, 3 and 4, and the percentage of Gal-1, integrin αv, integrin β3, and p-AKT -positive cells was also scored in four groups: 0 (0%), 1 (1 to 25%), 2 (26 to 50%), 3 (51 to 75%) and 4 (76 to 100%). The correlation analysis was performed among Gal-1 and integrin αv/ integrin β3/ p-AKT.
