# Supplementary material for: Galectin-1 induces hepatocellular carcinoma EMT and sorafenib resistance by activating FAK/PI3K/AKT signaling
Source: Cell Death Dis. 2016 Apr 21;7(4):e2201–. doi: 10.1038/cddis.2015.324 (PMC4855644; doi:10.1038/cddis.2015.324)
Supplement: Supplementary Table 1 [file cddis2015324x3.doc]

Supplementary Table 1. Correlation between Gal-1 and clinicopathological characteristics in 209 HCCs

**Abbreviations and Note:** HBsAg, hepatitis B surface antigen; BCLC, Barcelona-Clinic Liver Cancer.

| Variable | Gal-1 | | | | P value | | |  |
| --- | --- | --- | --- | --- | --- | --- | --- | --- |
|  | Low | | High | |  |  |  |  |
| Age (years) | | | | | | |  | |
| ≤50 | 38 | | 49 | | 0.25 | | |  |
| >50 | 43 | | 79 | |  |  |  |  |
| Sex | | | | | | |  | |
| Female | 15 | | 15 | | 0.224 | | |  |
| Male | 66 | | 133 | |  |  |  |  |
| HBsAg | | | | | | |  | |
| Negative | 18 | | 24 | | 0.596 | | |  |
| Positive | 63 | | 104 | |  |  |  |  |
| HCVAb | | | | | | | |  |
| Negative | 79 | | 128 | | 0.149 | | |  |
| Positive | 2 | | 0 | |  | | |  |
| Liver cirrhosis | | | | | | |  | |
| No | 9 | | 13 | | 0.821 | | |  |
| Yes | 72 | | 115 | |  |  |  |  |
| Serum AFP, ng/mL | | | | | | |  | |
| ≤20 | 36 | | 42 | | 0.1 | | |  |
| >20 | 45 | | 86 | |  |  |  |  |
| Serum ALT, U/L | | | | | | |  | |
| ≤75 | 69 | | 114 | | 0.519 | | |  |
| >75 | 12 | | 14 | |  |  |  |  |
| Tumor size (diameter, cm) | | | | | | |  | |
| ≤5 | 55 | | 74 | | 0.189 | | |  |
| >5 | 26 | | 54 | |  |  |  |  |
| Tumor encapsulation | | | | | | |  | |
| Absent | 45 | | 57 | | 0.155 | | |  |
| Present | 36 | | 71 | |  |  |  |  |
| Vascular invasion | | | | | | |  | |
| No | 63 | | 90 | | 0.246 | | |  |
| Yes | 18 | | 38 | |  |  |  |  |
| Tumor number | | | | | | |  | |
| Single | | 68 | | 107 | | 1.000 | |  |
| Multiple | | 13 | | 21 | |  |  |  |
| Differentiation | | | | | | |  | |
| I/II | | 60 | | 92 | | 0.753 | |  |
| III/IV | | 21 | | 36 | |  |  |  |
| BCLC staging | |  | |  | |  | |  |
| 0/A | | 65 | | 82 | | 0.013 | |  |
| B/C | | 16 | | 46 | |  | |  |
