# Supplementary material for: Galectin-1 induces hepatocellular carcinoma EMT and sorafenib resistance by activating FAK/PI3K/AKT signaling
Source: Cell Death Dis. 2016 Apr 21;7(4):e2201–. doi: 10.1038/cddis.2015.324 (PMC4855644; doi:10.1038/cddis.2015.324)
Supplement: Supplementary Table 2 [file cddis2015324x4.doc]

Supplementary Table 2. Univariate and Multivariate Analyses of Factors Associated with Overall Survival

|  | OS | | | |
| --- | --- | --- | --- | --- |
|  |  | Multivariate | | |
| Factors | Univariate, P | HR | 95% CI | P value |
| Sex (female vs. male) | 0.128 |  |  | NA |
| Age (years) (≤50 vs. >50) | 0.296 |  |  | NA |
| HBsAg (positive vs. negative) | 0.082 |  |  | NA |
| HCVAb (positive vs. negative) | 0.708 |  |  | NA |
| Child-Pugh classification (A vs. B) | 0.012 | 0.932 | 0.243-3.571 | NS |
| Liver cirrhosis (yes vs. no) | 0.251 |  |  | NA |
| Serum AFP, ng/mL (≤20 vs.>20) | 0.093 |  |  | NA |
| Serum ALT, U/L (≤75 vs. >75) | 0.469 |  |  | NA |
| Tumor size (diameter, cm) (>5 vs. ≤5) | <0.0001 | 2.162 | 1.409-3.318 | <0.0001 |
| Tumor number (multiple vs. single) | 0.03 | 1.63 | 1.095-2.426 | 0.016 |
| Tumor differentiation (III/IV vs. I/II.) | 0.209 |  |  | NA |
| BCLC staging (0/A vs. B/C) | 0.006 | 1.747 | 0.444-6.876 | NS |
| Gal-1 expression (high vs. low) | 0.006 | 0.821 | 0.932-1.942 | 0.029 |

OS, overall survival; NA, not adopted; NS, not significant; AFP, alpha-fetoprotein; HBsAg, hepatitis B surface antigen; 95%CI, 95% confidence interval; BCLC,Barcelona-Clinic Liver Cancer HR, hazard ratio; Cox proportional hazards regression model.
