# Supplementary material for: Galectin-1 induces hepatocellular carcinoma EMT and sorafenib resistance by activating FAK/PI3K/AKT signaling
Source: Cell Death Dis. 2016 Apr 21;7(4):e2201–. doi: 10.1038/cddis.2015.324 (PMC4855644; doi:10.1038/cddis.2015.324)
Supplement: Supplementary Table 3 [file cddis2015324x5.doc]

Supplementary Table 3. Univariate and Multivariate Analyses of Factors Associated with Cumulative Recurrence

|  | Cumulative Recurrence | | | |
| --- | --- | --- | --- | --- |
|  |  | Multivariate | | |
| Factors | Univariate, P | HR | 95% CI | P value |
| Sex (female vs. male) | 0.257 |  |  | NA |
| Age (years) (≤50 vs. >50) | 0.421 |  |  | NA |
| HBsAg (positive vs. negative) | 0.934 |  |  | NA |
| HCVAb (positive vs. negative) | 0.827 |  |  | NA |
| Child-Pugh classification (A vs. B) | 0.015 |  |  | NS |
| Liver cirrhosis (yes vs. no) | 0.322 |  |  | NA |
| Serum AFP, ng/mL (≤20 vs.>20) | 0.016 | 1.874 | 1.183-2.968 | 0.007 |
| Serum ALT, U/L (≤75 vs. >75) | 0.28 |  |  | NA |
| Tumor size (diameter, cm) (>5 vs. ≤5) | <0.0001 | 2.133 | 1.38-3.296 | 0.001 |
| Tumor number (multiple vs. single) | 0.015 | 1.792 | 1.208-2.661 | 0.004 |
| Tumor differentiation (III/IV vs. I/II.) | 0.376 |  |  | NA |
| BCLC staging (0/A vs. B/C) | 0.009 | 1.932 | 0.5-7.471 | NS |
| Gal-1 expression (high vs. low) | 0.008 | 0.612 | 0.599-1.677 | 0.078 |

NA, not adopted; NS, not significant; AFP, alpha-fetoprotein; HBsAg, hepatitis B surface antigen; 95%CI, 95% confidence interval; BCLC,Barcelona-Clinic Liver Cancer HR, hazard ratio; Cox proportional hazards regression model.
