# Supplementary material for: Galectin-1 induces hepatocellular carcinoma EMT and sorafenib resistance by activating FAK/PI3K/AKT signaling
Source: Cell Death Dis. 2016 Apr 21;7(4):e2201–. doi: 10.1038/cddis.2015.324 (PMC4855644; doi:10.1038/cddis.2015.324)
Supplement: Supplementary Table 4 [file cddis2015324x6.doc]

Supplementary Table 4. Demographic and Baseline Characteristics of the Patients (Sorafenib-Treated Population)

| Variable | Gal-1^low^ (N=15) | Gal-1^high^ (N=15) |
| --- | --- | --- |
| Age-yr | 47.12 ± 11.35 | 53.47 ± 9.32 |
| Sex-no | | |
| male | 13 | 14 |
| female | 2 | 1 |
| Cause of disease-no | | |
| Hepatitis B | 14 | 15 |
| other | 1 | 0 |
| BCLC stage-no (%) | | |
| B(intermediate) | 6 | 4 |
| C(advanced) | 9 | 11 |
| Child-Pugh class-no (%) | | |
| A | 9 | 14 |
| B | 6 | 1 |
| Biochemical analysis | | |
| Albumin-g/dl | | |
| Median | 4.1 | 4.9 |
| Range | 2.5-4.9 | 3.7-5.4 |
| Total bilirubin-μmol/L | | |
| Median | 15.2 | 11.1 |
| Range | 6.4-25.9 | 6.3-20.7 |
| Alpha-fetoprotein-ng/ml | | |
| Median | 348 | 72 |
| Range | 3.7-1.23×10^4^ | 1.6-1.7×10^4^ |

**Abbreviations and note:** BCLC, Barcelona-Clinic Liver Cancer.
