# Supplementary figures and images for: Galectin-1 induces hepatocellular carcinoma EMT and sorafenib resistance by activating FAK/PI3K/AKT signaling
Source: Cell Death Dis. 2016 Apr 21;7(4):e2201–. doi: 10.1038/cddis.2015.324 (PMC4855644; doi:10.1038/cddis.2015.324)

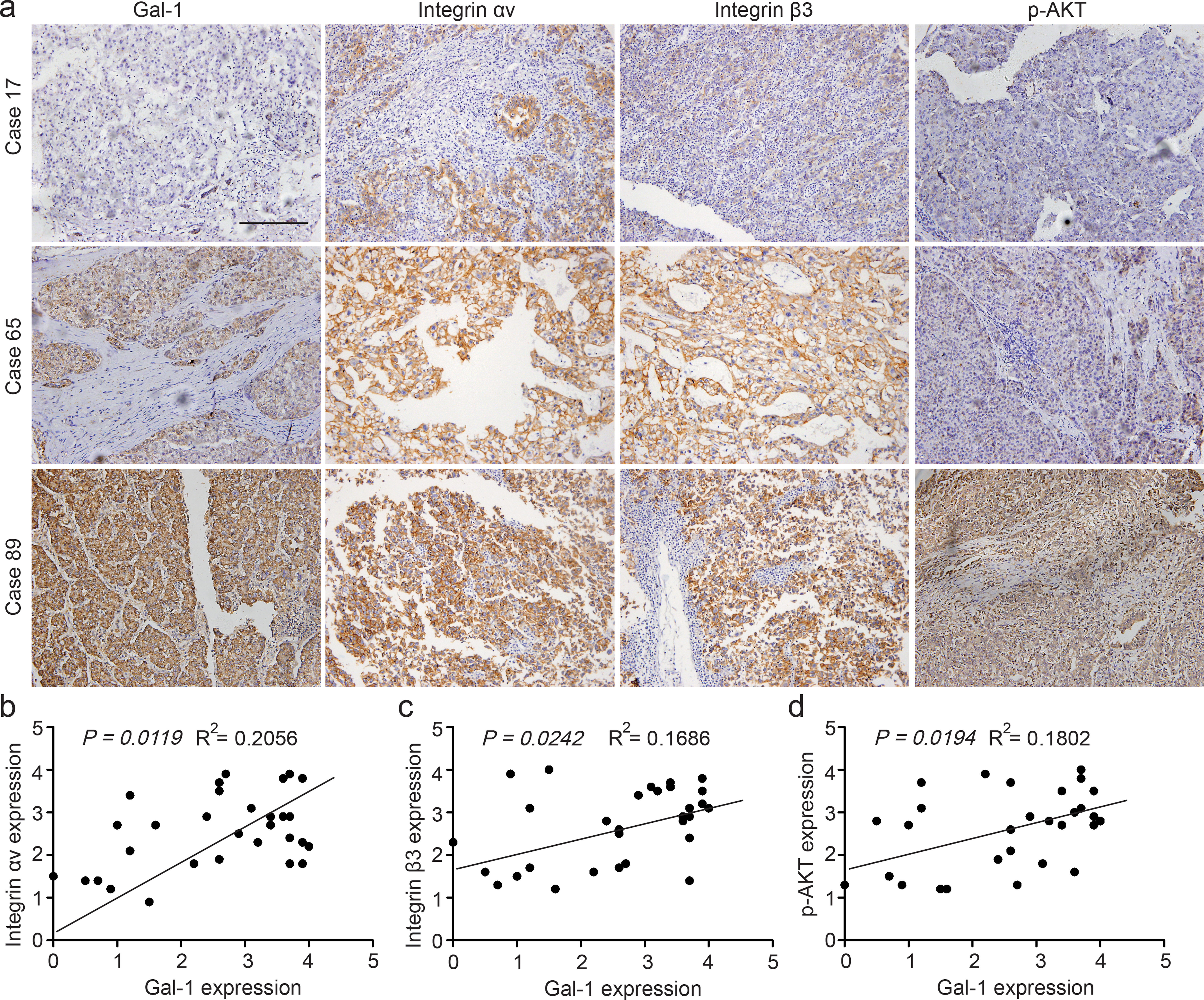

Supplement: Supplementary Figure 1 [file cddis2015324x7.tif]

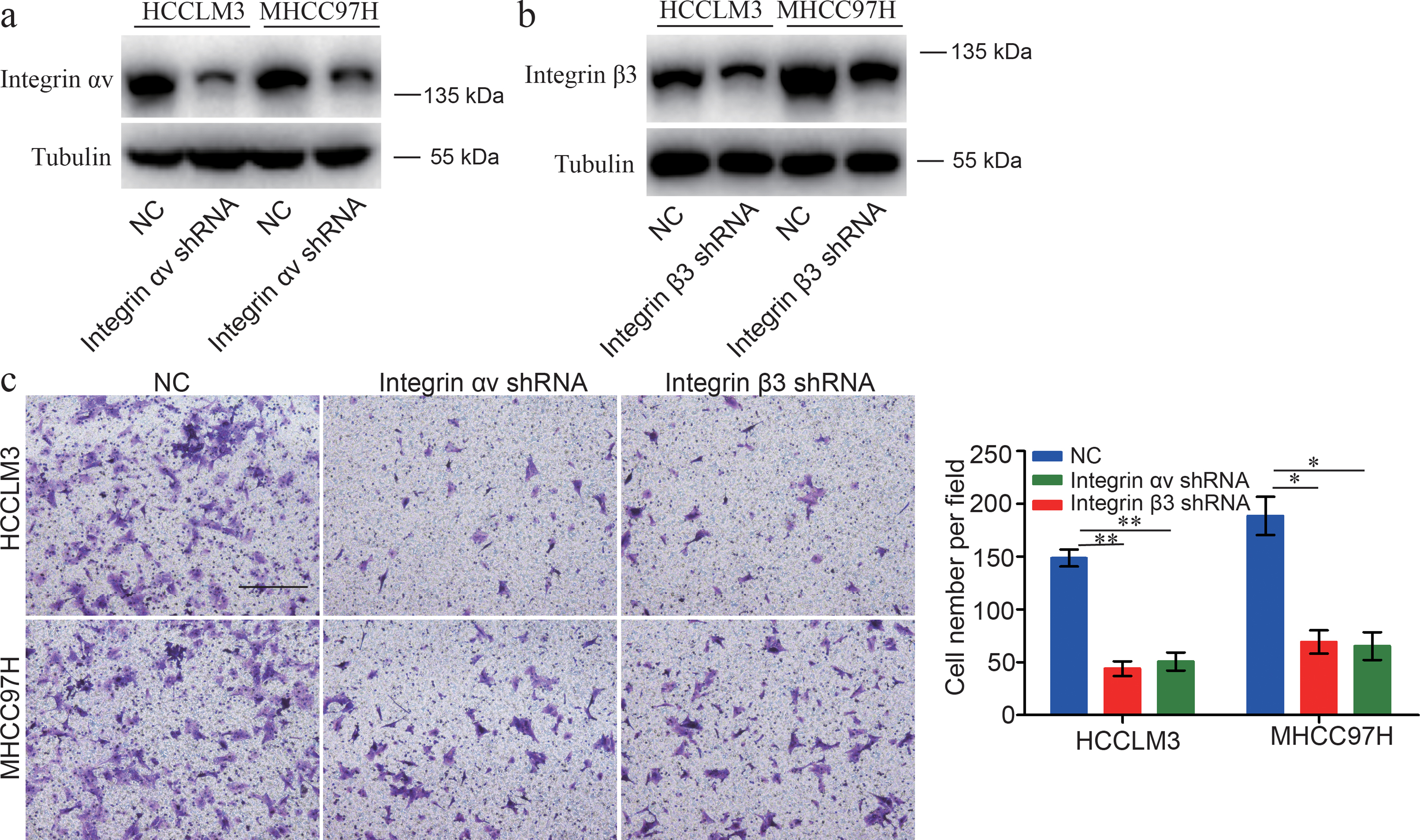

Supplement: Supplementary Figure 2 [file cddis2015324x8.tif]
